# Field and Laboratory Responses of Male Leaf Roller Moths, *Choristoneura rosaceana* and *Pandemis pyrusana*, to Pheromone Concentrations in an Attracticide Paste Formulation

**DOI:** 10.1673/031.009.4501

**Published:** 2009-06-24

**Authors:** Tomislav Curkovic, Jay F. Brunner, Peter J. Landolt

**Affiliations:** ^1^ Departamento de Sanidad Vegetal, Facultad de Ciencias Agronómicas, Universidad de Chile, Casilla 1004, Santiago; ^2^ Tree Fruit Research and Extension Center, Washington State University, Wenatchee, WA, 98801; ^3^ USDA-ARS Yakima Agricultural Research Laboratory, 5230 Konnowac Pass Road, Wapato, WA, 98951

**Keywords:** inhibitor threshold, septa, source contact, wind tunnel

## Abstract

Male leafroller moths, *Choristoneura rosaceana* (Harris) (Lepidoptera: Tortricidae) and *Pandemis pyrusana* (Kearfott), were evaluated for responses to a paste formulation loaded with a range of concentrations of the two species' pheromone blends and evaluated in a laboratory wind tunnel and in the field. Response criteria were flight, flight towards the pheromone source, and contact with the pheromone source for the wind tunnel assays, and capture of moths in traps for the field tests. In the wind tunnel and field, responses of males of both species to the paste generally increased as the pheromone concentration in the paste was increased. There was little response by either species to paste with less than 0.16% pheromone. The relationship between pheromone concentration and response for *P. pyrusana* was linear and for *C. rosaceana* was sinusoidal over the range of pheromone concentrations tested. These patterns were seen both in the wind tunnel and in the field. Initial release rates from the paste of (*Z*)-11-tetradecenyl acetate, the main component of the pheromone blends of both species was 3.6–3.8 ng/h. Inhibitory thresholds for responses were not reached for either species, using pheromone concentrations as high as 16%, in either the wind tunnel or the field. For both species, response of males to rubber septa with one mg pheromone loads was similar to the response to the paste with pheromone at concentrations greater than 3–4%. For *C. rosaceana*, rates of contact with the paste in the wind tunnel were statistically similar to rates of contact in response to conspecific females, with paste pheromone concentrations above 1.6%. Response rates for males of *P. pyrusana* were significantly lower to the paste than to conspecific females at all paste pheromone concentrations tested. Overall, the optimum pheromone concentration in the paste for moth attraction to contact was 3.2 % for *C. rosaceana* and 8% for *P. pyrusana*.

## Introduction

Insect communication mediated by sex attractant pheromones depends on exposure to appropriate atmospheric concentrations of pheromone. Such concentrations of pheromone in air depend in part on initial rates of evaporation at the source, wind speed, and patterns of diffusion, all of which are affected by environmental conditions (Flint et al. 1990; Zeoli et al. 1982; Bierl-Leonhardt 1982). Many studies of insect responses to attractive sex pheromones correlate the amount or dose of pheromone to the level of response (Carde and Charlton 1984), although this is removed from the parameters more directly impacting olfaction and behavioral responses. However, with formulation methods in use for most insect pheromone studies, dose does affect evaporation rate, which affects concentration in air.

Insects can assess relative concentrations of pheromone in air and may respond differently to varied concentrations. For example, high concentrations may indicate proximity to the source and trigger behavioral changes (Boeckh 1984). Hagaman and Carde (1984) and McNeil et al. (1997) showed that virgin females produce more pheromone than mated females, and male moths may then respond better to higher pheromone concentrations as a way to increase their reproductive fitness. That may contribute to the tendency for a higher pheromone evaporation rate at a source to attract more males (Sans et al. 1997). In Lepidoptera a behavioral response is initiated at some relatively low pheromone concentration (activation threshold) and termination of that behavior occurs at a higher concentration (inhibitory threshold) (Roelofs 1978). The range of pheromone concentrations between the activation and inhibitory thresholds is defined as an “attraction area”, where males respond. The pattern of responses may follow an s-shaped or a linear relationship over increasing pheromone doses or concentrations (Kamm and McDonough 1979, Kamm et al. 1989).

Attracticides are baits that combine attractants such as synthetic pheromones and an insecticide to attract and then kill the target species (Charmillot et al. 2000). These baits are also referred to as attract-and-kill or lure-and-kill. The technology represents a specific alternative to conventional application of insecticides (Hofer and Angst 1995) and offers a means to reduce amounts of insecticides used as well as insecticide contact with the crop, the environment and non-target insects. Field demonstrations of reductions of pest populations with attracticides have been reported for several species of tortricid moths, e.g. *Chonstoneura rosaceana* (Curkovic and Brunner 2007), *Cydia molesta* (Evenden and McLaughlin 2005), *C. pomonella* (Charmillot et al. 1996), *Epiphyas postvittana* (Suckling and Brockerhoff 1999), and *Pandemis pyrusana* (Curkovic and Brunner 2007).

An optimized concentration of an attractive pheromone lures the maximum number of males to the source while eliciting a high level of source contact (Carde and Minks 1995), a mode of action required for attracticides (Krupke 1999; Curkovic and Brunner 2005). It is possible to evaluate attraction to pheromone lures by comparing male moth captures in traps baited with different pheromone sources, including different attracticide formulations. However, this kind of evaluation does not show contact with the pheromone source, which is required for attracticide efficacy (Vickers and Rothschild 1991; Campion et al. 1980). Therefore, we used both wind tunnel and field experiments to evaluate a paste formulation used for attracticide products against pest Tortricidae. The principal objective of this study was to determine the pheromone release rate from the paste that maximizes both attraction and contact by *C. rosaceana* and *P. pyrusana* males to their respective sex pheromones.

## Materials and Methods

### Insects

*Choristoneura rosaceana* and *P. pyrusana* pupae were obtained from colonies maintained as described by Curkovic (2004). Colonies were maintained in walk-in growth chambers at 23 ± 2 °C and 45 ± 5% RH, under a photoperiod regime of 16:8 h (L:D). Pupae were sexed, washed in 0.05% chlorine bleach solution, placed in groups of 15–20 per plastic cup, provided with honey water solution, and kept under the same temperature, humidity, and lighting conditions. Male and female pupae were held in different chambers to avoid moth pre-exposure to con-specific sex pheromone. Upon emergence, unisexual groups of 4–5 adults were placed in plastic cups and provided with a honey solution via cotton wicks. Two to four day-old males and two to six day-old females were used in experiments since they are sexually mature at those ages (Delisle 1995).

### Wind tunnel

The wind tunnel and conditions during bioassays were described by Curkovic and Brunner (2006). The airspeed was set at ca. 45 cm/s in the middle of the tunnel. Relative humidity in the room was 55±5% RH and light intensity was ca 2 lux. Female moths in cages, or synthetic pheromone sources, were placed on a horizontal platform held by a ring stand ca 10 cm downwind of the center of the upwind end of the wind tunnel. Synthetic pheromone sources were either paste droplets or rubber septa loaded with pheromone, placed inside a hair roller (L and N Sales and Marketing), which consisted of a tubular plastic frame covered with plastic mesh (6 cm length, 1.5 diameter) that was closed on each end with a Styrofoam lid. Cages with male moths were placed on a second platform near the center of the downwind end of the tunnel, ca. 70 cm from the platform holding the female moths or pheromone lure. The inside surfaces of the wind tunnel, plastic platforms, mesh moth cages, stands, and clamps were cleaned prior to each experiment. The Plexiglas surfaces of the wind tunnel were cleaned with 70% ethanol prior to every experiment. All moth containers, movable platforms, stands, and clamps were rinsed with 5% bleach solution, 70% ethanol, and baked at 200 °F for 2 h before additional experiments were run. Also air was allowed to flow through the wind tunnel for at least 12 h to remove any possible pheromone contamination before experiments were continued. Plastic gloves were used all the time during sources and males manipulation in the wind tunnel room.

### Pheromones and stock solutions

Pheromone components used in 2001 trials were analyzed by gas chromatography to determine purity. Purity of pheromone components used in 2002 was provided by the manufacturer (Bedoukian Research Inc., www.bedoukian.com). In all cases purity was >95%. In 2001, concentrated stock solutions were made of the pheromones of *C. rosaceana* and *P. pyrusana*, which were then diluted in hexane to obtain different concentrations for use in loading lures. The pheromone blend for *C. rosaceana* was a 95.5:2:1.5:1 ratio of (*Z*)-11-tetradecenyl acetate (Z11-14:Ac): (*E*)-11-tetradecenyl acetate (E11-14Ac): (*Z*)-11-tetradecenol (*Z*11-14OH): (*Z*)-11-tetradecenal (*Z*11-14Al) (Vakenti et al. 1988). For *P. pyrusana*, this was a 94:6 ratio of *Z*11-14Ac: *Z*9-14Ac (Roelofs et al. 1977). Pheromone solutions were stored at - 14° C, then held for 30 min at room temperature (24 ± 1° C) before mixing with the paste. No pheromone stock solutions were prepared in 2002 because Advanced Pheromone Technologies (http://advancedpheromonetech.com) prepared the paste with pheromone.

### Mixture procedure

The material used in this study was a paste used in the commercial formulation of the attracticide Last Call (Advanced Pheromone Technologies). This paste included an ultraviolet stabilizer (70% of the paste), a thickener, and a sticker. The paste was mixed with pheromone or was used alone as an experimental control. Pheromone was added to the paste by two procedures. In 2001 the stock pheromone solution was placed on a digital scale in a fume hood for 30 minutes to allow hexane evaporation. The paste was injected into vials containing the pheromone stock solutions until the necessary amount by weight was obtained to provide the desired concentration. Then the paste and pheromone were mixed manually using a glass stir rod while the vial was kept in a warm water bath (45 ± 5° C). This provided a paste with the highest pheromone concentration of 4%. The paste containing pheromone was then diluted by adding a measured amount of additional paste to obtain lower pheromone concentrations. Paste with the different pheromone concentrations desired were placed into labeled syringes and stored in a cold room (0° C). Syringes were placed at room temperature (24 ± 1° C) for 15–30 minutes before the paste was used in experiments. In 2002, larger amounts of paste were prepared Advanced Pheromone Technology following a procedure as described above, except that the pheromones were directly added to the paste by using a micro syringe and mixing with a mechanical stirrer.

### Handling and acclimation of moths for wind tunnel assays

Male moths were placed individually into a 4 × 2 cm mesh cylinder cage covered with a plastic lid on top and provided with honey solution via a cotton wick. Moths in cages were placed on a table next to the wind tunnel for 2–3 h before experiments. Trials were run in the first 2–4 hours of the scotophase, which corresponds to the sexual activity period for both *C. rosaceana* and *P. pyrusana* (Evenden 1998; Knight et al. 1996; Knight and Turner 1998). A pheromone source (paste, rubber septum, or female moth) was placed on the source platform and a caged male moth was then set on the downwind release platform of the wind tunnel. After two minutes the plastic lid was removed from the top of the male cage. A female moth used as an attractive source was placed in a 4 × 3 cm mesh cylinder cage and kept in a fume hood in an adjacent room for 1 h before an experiment. The female moths were then transferred to the wind tunnel in a sealed plastic bag that was opened inside the wind tunnel. The cage with female moth was placed on the upwind platform. To confirm that females were calling they were illuminated with a flashlight covered with red filter (Kodak gelatin #29, www.kodak.com). Only females showing the characteristic calling posture were assayed as attractant sources.

### Observation of moth behavior in wind tunnel

Visual observations in wind tunnel bioassays were made after 10 minutes of observer acclimation to the low light levels and by using a flashlight with a red filter. Within a 10 min assay period, male moth responses to pheromone sources were categorized as flying out of the cage, flying towards the pheromone source, and making contact with either the cage containing a female or hair roller containing either paste or rubber septum. A vacuum device (Bioquip #2820 A y B, www.BioQuip.com) was used to remove each male from the wind tunnel following the assay.

### Field tests

Pheromone lures for *C. rosaceana* were 1 mg of the 4-component blend in red rubber septa (Advanced Pheromone Technologies). Pheromone lures for *P. pyrusana* were 1.5 mg of the 2 component blend in red rubber septa (Suterra, www.suterra.com). The paste was evaluated as a 50 *µl* drop on a piece of aluminum foil inside a hair roller sealed with styrofoam plugs at both ends. These devices were pinned inside Delta traps (Suterra, www.suterra.com). Traps with septa or the paste were placed in trees in an apple orchard at a height of 1.5–2 m and at least 30 m between traps. Traps were checked every two to three days to record and remove captured moths and replace sticky bottoms as needed. Traps were rotated clockwise after each examination to minimize impact of trap location on moth captures. Field trials were established in Washington apple orchards infested with *C. rosaceana* and *P. pyrusana*. These orchards were not sprayed with insecticides during the period of trials.

**Figure 1.**
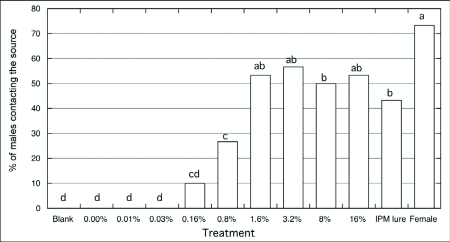
Percentage of source contact of *Choristoneura rosaceana* males (n = 30/treatment) to different pheromone concentrations (0 – 16%) at the paste source vs. septa and calling female, indoor wind tunnel trials, χ^2^ and SNK test (p = 0.05).

### Experimental Design and Data Analysis

For wind tunnel experiments a χ^2^ test (p = 0.05) was used to analyze categorical data with an adaptation of the SNK test to identify multiple differences between proportions of *C. rosaceana* and *P. pyrusana* males responding to pheromone sources. One treatment was run per night (n = 30/treatment). The SE value was 3.68 for both species, based on Zar (1996). P-values were estimated from χ^2^ tables. For field experiments, data for captures of *C. rosaceana* and *P. pyrusana* males in traps were analyzed by ANOVA and SNK test (Zar 1996). Treatments in orchard field trials were randomly blocked (n = 3 or 4 blocks). Proportions of cumulative moth captures within blocks were transformed by arcsine square root (Krupke 1999). All graphs with bars include SEM values. P-values were estimated from F tables.

## Results and Discussion

### Choristoneura rosaceana

#### Wind tunnel assays

Male *C. rosaceana* did not respond to the paste with no or with the lowest pheromone load tested (0–0.03%) (Figure 1). No males flew toward these sources. The statistical analysis for behavioral sequences indicated that the attractive paste with the lowest pheromone concentrations resulted in a significantly higher proportion of males staying on the release platform compared to other pheromone concentrations in the paste (χ^2^ = 342.82; p < 0.001). Male flight towards the paste and source contact was not observed until pheromone concentration in the attractive paste was higher than 0.16%. However, source contact was not statistically different between this lower concentration and the next greater concentration of 0.8%. Source contact increased significantly (χ^2^ = 157.14; p < 0.001) when the pheromone concentration in the paste was 1.6% or greater, but with no statistical differences between concentrations of 1.6% and 16%. The highest level of source contact occurred with male response to calling females. Male source contact in response to females was significantly higher than to pheromone lures (septa) and to the 8% pheromone in paste, but was not significantly different from 1.6, 3.2, and 16% pheromone in paste. Sweeney and McLean (1990) observed that male spruce budworm, *Choristoneura fumiferana*, response to calling females was higher than to several doses of the pheromone. The overall pattern of response in *C. rosaceana* was an s-shaped curve (Figure 1), where males responded to increasing pheromone concentrations up to some maximum level after which response remained similar (Kamm et al. 1989). Similar patterns of responses to a range of pheromone doses or concentrations have been observed with other Lepidoptera (Baker and Roelofs 1981: Bellas and Bartell 1983).

**Figure 2.**
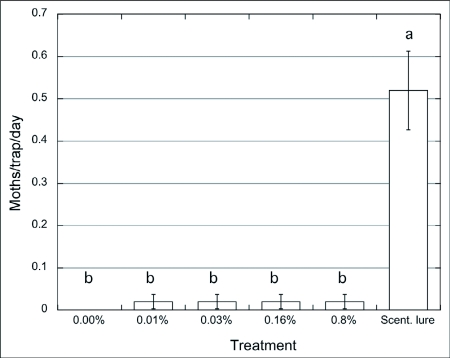
Captures of males of *Choristoneura rosaceana*/trap/day (n = 3) baited with paste + pheromone blend (0 – 0.8%) vs. septa, Wenatchee, WA, Aug. 12 – 28/2001. ANOVA and SNK (p = 0.05).

The range of pheromone concentrations in the paste tested in the wind tunnel covered 4 orders of magnitude. Sweeney and McLean (1990) evaluated synthetic *C. fumiferana* pheromone over a pheromone concentration range of 4 orders of magnitude, and found significant differences only between the extreme concentrations (0.00005% vs. 0.5%). Our results suggest that the activation threshold for the attractive paste formulation is between 0.03% and 0.16% pheromone concentration in paste. The positive response covered at least 3 orders of magnitude of pheromone concentration (0.16 to 16%), also observed by Mankin et al. (1980) for *Plodia interpunctella*, and Sweeney and McLean (1990) for *C. fumiferana*. No inhibitory threshold was reached as there was no reduction in response, even at the highest pheromone concentration tested (16%). McNeil et al. (1997) reported that young and non-mated moth females release larger amounts of pheromones. Thus, it is possible that the type of response we observed occurred because *C. rosaceana* males increase their fitness if they follow plumes that originate from sources with larger amounts of pheromone. The behavioral sequence during the approach of *C. rosaceana* males to a single pheromone source (loaded with different pheromone concentrations) was identical among them, and also to the sequence previously reported by Curkovic et al. (2006).

### Field trials

**Figure 3.**
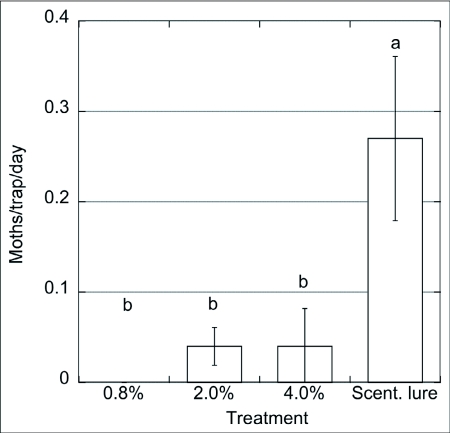
Captures of males of *Choristoneura rosaceana*/trap/day (n = 3) baited with paste + pheromone blend (0.8 – 4%) vs. septa, Wenatchee, WA, Sep. 1 – 16/2001. ANOVA and SNK (p = 0.05).

In 2001, traps baited with the paste with no pheromone (control) did not capture wild male *C. rosaceana* (Figure 2). While a small number of males were captured in traps baited with paste containing low pheromone concentrations there were no statistical difference between them and control traps. None of the paste treatments were nearly as attractive as the rubber septa that showed significantly greater captures of male moths (F = 16.97; p < 0.001). Another trial (Figure 3) included pastes with higher concentrations of pheromone, 0.8 – 4%. In this trial traps with the lower concentration (0.8%) did not capture any moths whereas traps with this same treatment captured some moths in the previous experiment (Figure 2). There were no significant differences between traps baited with the paste with the lowest (0.8%) or highest (4%) concentration although moths were captured only in traps baited with paste with the two higher concentrations of pheromone. The traps baited with the septa captured significantly more moths than traps with any of the paste treatments (F = 6.37; p < 0.028). These observations suggest that males might be able to identify, and differentially choose and approach, sources when exposed to different pheromone amounts, as observed by Mafra-Neto and Baker (1996). These authors found that almond moth males, *Ephestia cautella*, preferred and contacted lures loaded with 50 ng of pheromone, then shifted their preference to 500 ng loaded lures after they were pre-exposed to a high concentration, presumably because adaptation/habituation was overcome by higher emission rates from the pheromone sources. Male moth captures using the highest concentration of pheromone in the paste (0.8% in Figure 2, and 4% in Figure 3) were ∼ 30x and ∼ 6x lower, respectively, than traps using the septa. Suckling and Brockerhoff (1999) evaluated male captures of *E. postvittana* in traps baited with 100- µl droplets of an attracticide (similar to the paste we used) loaded with 300 and 3,000 µg of pheromone vs. commercial lures (100 µg/septum). Unlike our results, they found no differences using an attracticide paste loaded with 3 to 30 times the amount of pheromone contained in the septa, obtaining male captures ranging from 0.5 (300 µg) to 2x (3 mg) the numbers captured in traps baited with septa. In our experiments only the highest pheromone concentration, i.e. 4% (or 2 mg of pheromone) approximated the attractiveness of the pheromone dose provided in the septa lures. It is possible that the septa lures released more pheromonethan the paste even though they were loaded with similar amounts of pheromone.

**Figure 4.**
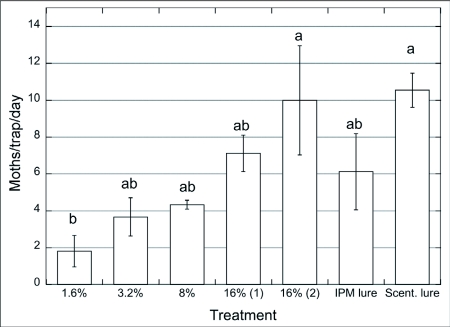
Captures of males of *Choristoneura rosaceana*/trap/day (n = 4) baited with paste + pheromone blend (1.6 – 16%, one droplet/trap; 16%, two droplets/trap) vs. septa, Winchester, WA, Jun. 15 – 26/2002. ANOVA and SNK test (p = 0.05).

Release of pheromone from lures has been shown to follow a first order kinetic pattern (Butler and McDonough 1981), i.e. the release rate is proportional to the remaining amount of pheromone, and therefore the release is high at the beginning but becomes lower over time (Zeoli et al. 1982). On the other hand, Kirsch (2000) indicated that the paste released codlemone at a constant rate the first 4 weeks, apparently following zero order kinetics (Zeoli et al. 1982). This indicates that the paste would have a slower rate of release of pheromone than a septum, at least initially. Curkovic and Brunner (2003) reported significantly greater attraction of *Cydia pomonella* males to septa pheromone lures compared to a pheromone-baited paste over a 3-week period. This suggests that lures using a paste-like formulation might be very long lasting and useful in monitoring for longer periods than septa. If it is assumed that a comparable pheromone release rate behavior between the codling moth and leafroller pheromone components in both lure formulations, it would be expected that the paste would attract fewer males but have a longer lasting activity than the lure. Our field trials were probably not run for long enough periods to fully evaluate such a hypothesis, in fact none lasted longer than 4 weeks. Alternatively, if the pheromone release rates were similar between septa and paste, differences in attraction might have been due to either differential release of pheromone components, absence of release of some pheromone components, or other unknown factors. Release rates have been shown to vary depending on chemical features of pheromone components (Quisumbing and Kydonieus 1982). Release rate of Z11-14Ac from the 1.6% paste formulation was estimated initially to be 3.6 ng/h for the *C. rosaceana* blend and 3.8 ng/h for the *P. pyrusana* blend (Peter J. Landolt, unpublished data). This is close to the minimum release rate range of 4 to 20 ng/h reported for virgin *C. fumiferana* females under airflow (Ramaswamy and Cardé 1983) and much lower than reported Z11-14Ac release rates from septa (Butler and McDonough 1981). These data agree with the wind tunnel results that showed similar male responses to calling females and to the attracticide paste with a 1.6% pheromone concentration or above.

In a 2002 final field trial (Winchester, WA) a paste was used that contained higher concentrations (1.6 to 16%) of *C. rosaceana* pheromone and two different kinds of commercial pheromone lures. Moth captures in traps baited with the lowest paste pheromone concentration (1.6%) were significantly lower than in traps baited with the highest paste pheromone concentration (16% pheromone with two drops) and in traps baited with the Suterra septa (Figure 4; F = 13.89; p < 0.001). These data confirm results from indoor wind tunnel assays, i.e. *C. rosaceana* males approached the paste only when loaded with the highest concentrations of pheromone. Stelinski et al. (2007) also found that males of *C. rosaceana* from three US states showed an increased response to pheromone sources with increasing pheromone quantities. This finding suggests that the upper range of pheromone concentrations that males were exposed to in our field trials were lower than the pheromone concentrations that caused adaptation of *C. rosaceana* antennae (Trimble and Marshall 2007) following atomization of a Z11-14Ac solution in the laboratory. Another factor possibly influencing observed results is the active space of the pheromone source. Baker and Roelofs (1981) indicated that a higher pheromone concentration in a source increased the “active distance” or the maximum drawing distance of males to the source and therefore the final number of individuals captured in traps. However, it would be possible for lure efficiency to decrease at very high pheromone concentrations or doses at the source when numerous males are recruited downwind but an inhibitory threshold is reached preventing moths from contacting the source. This was probably not the case in our field trials since moth captures showed an increase over the whole range of pheromone concentrations tested (Figure 4). Lure aging effects are also of concern of course. As pheromone lures age they tend to become less attractive (Leonhardt et al. 1990) which could be responsible for reducing the active space and the duration over which the lure was attractive.

**Figure 5.**
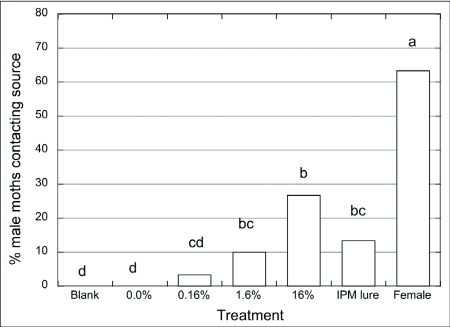
Percentage of source contact of *Pandemis pyrusana* males (n = 30/treatment) to different pheromone concentrations (0 – 16%) at the paste source vs. septa and calling female, indoor wind tunnel trials, χ^2^ and SNK test (p = 0.05).

### Pandemis pyrusana

#### Wind tunnel assays

*Pandemis pyrusana* males did not respond to the paste that was without pheromone (control). The lowest paste pheromone concentration tested was 0.16% and *P. pyrusana* males made source contact with this pheromone concentration as well as with all higher concentrations, but with a significant difference in the proportion of males making contact between the 0.16% and 16% treatment (χ^2^ = 37.71; p < 0.001) (Figure 5). The highest level of source contact by males occurred with the calling *P. pyrusana* female. There were no differences between male contact with the septa and any of the paste treatments (0.16% pheromone or higher). When compared to *C. rosaceana* (Figure 1) the percent of males making source contact with the paste was at least 2 times lower for *P. pyrusana* at the same paste pheromone concentration (Figure 5). These results suggest that the pheromone concentration, blend, or component ratios used for *P. pyrusana* were less attractive to male *P. pyrusana* than the *C. rosaceana* pheromone lures were to male *C. rosaceana*. It is possible that the methodology used (wind tunnel) could have been in part responsible for the low level of response in *P. pyrusana*, but the fact that the response to calling females in both species was relatively high (63–73%) implies that the impact of the bioassay environment was not greatly different between species. It is possible that the pheromone blend or ratio of components used for *P. pyrusana* was suboptimal, or that the paste formulation had some negative effect on the lure components. Alternatively, Daterman (1982) suggested that different species respond differentially to pheromone concentration and this might explain the differential results observed between *P. pyrusana* and *C. rosaceana* males in our tests. DeLury et al. (2006) reported that *P. limitata*, a closely related species to *P. pyrusana*, is monitored with the published blend that was used in this study. It is possible also that the pheromone identified for *P. pyrusana* is incomplete which could explain the relatively low attractancy of males exposed to the paste and to the septa. The behavior of *P. pyrusana* males approaching pheromone sources was identical to the sequence previously reported by Curkovic et al. (2006).

**Figure 6.**
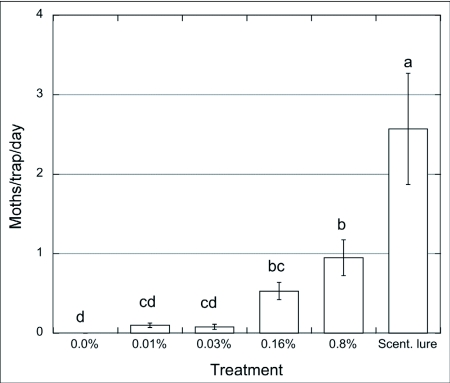
Captures of males of *Pandemis pyrusana*/trap/day (n = 3) baited with paste + pheromone blend (0 – 0.8%) vs. septa, Wenatchee, WA, Aug. 09 – 29/2001. ANOVA and SNK test (p = 0.05).

The response of *P. pyrusana* to the paste as a pheromone source increased significantly with increased pheromone concentration, with no leveling off of source contact as observed with *C. rosaceana* (Figure 5). These results indicate that any response threshold is above the 16% pheromone concentration tested for *P. pyrusana*. The absence of a reduction in males approaching and contacting the paste loaded with the highest pheromone concentration is consistent with reports of difficulties in managing these leafroller species with pheromonal mating disruption (Nobbs et al. 1999; Agnello et al. 1996). This may be due to a failure to produce atmospheric pheromone concentrations sufficient to reach an inhibitory threshold. The overall response pattern for *P. pyrusana* shows a linear and positive relationship between pheromone concentration and male attraction to the pheromone treated paste, similar to that observed for the peach twig borer, *Anarsia lineatella*, by Hathaway (1981).

**Figure 7.**
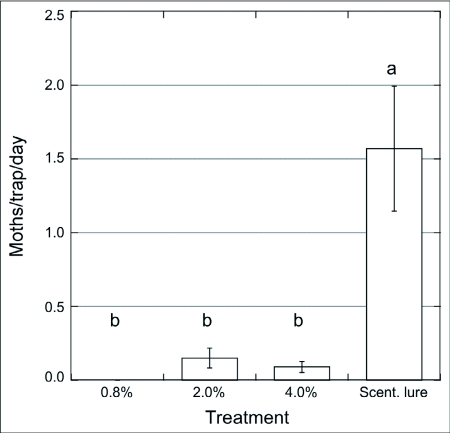
Captures of males of *Pandemis pyrusana*/trap/day (n = 3) baited with paste + pheromone blend (0.8 – 4%) vs. septa, Wenatchee, WA, Sep. 1 – 18/2001. ANOVA and SNK test (p = 0.05).

### Field trials

*Pandemis pyrusana* males were not captured in traps containing only the paste (no pheromone, Figure 6). There was a very low level of moth capture in traps baited with paste containing less than 0.16% pheromone and captures increased significantly as the concentration of pheromone increased to 0.16% and 0.8% (F = 11.75; p < 0.001). The greatest numbers of males captured with paste were in traps baited with paste loaded with the highest pheromone concentration (0.8%), although this was statistically similar to the 0.16% treatment. Septa baited traps captured significantly more male moths than traps with any of the paste treatments (F = 34.99; p < 0.001). In a later experiment in 2001, *P. pyrusana* captures in septa-baited traps were significantly greater than in traps baited with paste droplets loaded with up to 4% pheromone but with no differences among moth captures in traps with any of the paste treatments (Figure 7). There were no or very low captures of *P. pyrusana* in the paste treatments with lower pheromone concentrations in these two field trials (Figure 6, 0%–0.8% pheromone and Figure 7, 0.8%–4% pheromone). In addition, the highest moth captures occurred with the highest concentration in both trials, though the highest pheromone concentration was different in each. The discussion concerning the attraction of *C. rosaceana* males to higher pheromone sources (Figures 2 and 3), and the suggestion that males can choose between varied concentrations of pheromone from sources under field conditions, might also apply here to *P. pyrusana*. This hypothesis would agree with the common observation that traps baited with high-load lures (10x) for both species, *P. pyrusana* and *C. rosaceana*, capture more males than standard-load lures (IX) in orchards treated with the mating disruption. From the point of view of natural history this might also be related to a preference for virgin or larger females (Andersson and Iwasa 1996, McNeil et al. 1997).

**Figure 8.**
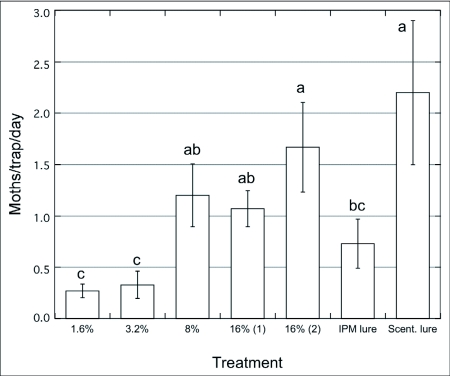
Captures of males of *Pandemis pyrusana*/trap/day n = 3) baited with paste + pheromone blend (1.6 – 16%; 16% with 1 or 2 droplets/trap) vs. septa, Winchester, WA, Jun. 15 – 26/2002. ANOVA and SNK test (p = 0.05).

In 2002 trials with *P. pyrusana* at Winchester showed significant differences between paste treatments. The formulation with the lowest pheromone concentrations, 1.6% and 3.2%, captured similar numbers of moths and these were statistically less than the paste treatments with 8% and 16% pheromone (Figure 8 ; F = 10.98; p < 0.001). Traps baited with the Suterra pheromone lures captured more males than any other treatment, including traps baited with the Advanced Pheromone Technologies pheromone lures. Numbers of males in traps baited with 8% pheromone in paste were statistically similar to numbers trapped with the Suterra lures. Considering that the statistical analysis was conducted on average cumulative captures, it is possible that the active space, residual attraction or aging effect, or a combination of these factors affected the final results, as discussed previously. These results also suggest that no inhibitory threshold was reached under these conditions.

This study was designed to provide basic information on moth responses to pheromone sources in order to evaluate the possibility of developing specific attracticides against both *C. rosaceana* and *P. pyrusana* males. The effect of pheromone concentration using a paste formulation on male behavior was examined, particularly source contact. Results from wind tunnel assays indicate that maximum source contact was obtained with pheromone concentrations above 1.6% for both species, and that no significant differences were observed when higher pheromone concentrations were evaluated. However, field trials showed that male responses (trap captures) increased with pheromone concentrations higher than 1.6% for both species, leveling off at 3.2% for *C. rosaceana* and 8% for *P. pyrusana*. Differences between indoor and field trials might be related to the “attractive area” hypothesis, which applies in the orchard-scale (i.e. larger pheromone concentrations attracting males from larger areas during a period of several days) but not in the wind tunnel-scale where the space is highly reduced and the evaluation does not consider possible aging effects of the source. These findings also open the opportunity to use this type of formulation for the development of new lures since they seem to provide longer lasting activity with regard to the septa, at low cost. In conclusion, it was demonstrated in the laboratory that both species increasingly responded to, and contacted, paste sources as pheromone concentrations increase over a certain threshold level. In the field males exposed to different sources chose pheromone sources with the highest loads. Based on these results it would be important to use a high pheromone concentration in a paste formulation in order to optimize attraction and competition with females under field conditions.
